# T-cell receptor-engineered T cells for cancer treatment: current status and future directions

**DOI:** 10.1007/s13238-016-0367-1

**Published:** 2017-01-20

**Authors:** Yu Ping, Chaojun Liu, Yi Zhang

**Affiliations:** 1 Biotherapy Center, The First Affiliated Hospital of Zhengzhou University, Zhengzhou, 450052 China; 2 Cancer Center, The First Affiliated Hospital of Zhengzhou University, Zhengzhou, 450052 China; 3 Engineering Key Laboratory for Cell Therapy of Henan Province, Zhengzhou, 450052 China

**Keywords:** T-cell receptor, tumor antigen, TCR-engineered T cells, neoantigen, tumor microenvironment

## Abstract

T-cell receptor (TCR)-engineered T cells are a novel option for adoptive cell therapy used for the treatment of several advanced forms of cancer. Work using TCR-engineered T cells began more than two decades ago, with numerous preclinical studies showing that such cells could mediate tumor lysis and eradication. The success of these trials provided the foundation for clinical trials, including recent clinical successes using TCR-engineered T cells to target New York esophageal squamous cell carcinoma (NY-ESO-1). These successes demonstrate the potential of this approach to treat cancer. In this review, we provide a perspective on the current and future applications of TCR-engineered T cells for the treatment of cancer. Our summary focuses on TCR activation and both pre-clinical and clinical applications of TCR-engineered T cells. We also discuss how to enhance the function of TCR-engineered T cells and prolong their longevity in the tumor microenvironment.

## INTRODUCTION

It was shown as early as 1976 that interleukin-2 (IL-2), regarded as a T-cell growth factor, induced T-cell proliferation without loss of effector function *in vitro* (Morgan et al., 1976). It is now known that the cytokine IL-2 is crucial for sustained clonal expansion of responding T cells. A study by Rosenberg et al. demonstrated that syngeneic tumor-infiltrating lymphocytes (TILs) could undergo expansion in the presence of IL-2. Adoptive transfer of these TILs to murine models was shown to lead to regression in lung and liver tumors (Rosenberg et al., 1986). Subsequently, adoptive cell therapy using autologous TILs has become one of the most effective approaches to induce long-lasting regression in patients with metastatic melanoma (Rosenberg et al., 1988; Dudley et al., 2002; Scanlan et al., 2002; Rosenberg et al., 2011; Pilon-Thomas et al., 2012; Radvanyi et al., 2012; Besser et al., 2013). The presence of TILs has also been associated with improved prognosis in other cancer types, including ovarian, colon, and breast cancer (Clemente et al., 1996; Sato et al., 2005; Galon et al., 2006; Loi, 2013).

**Figure 1:**
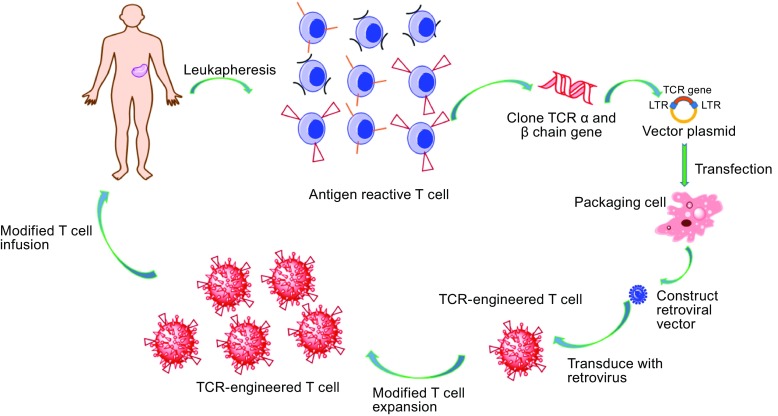
**Process of TCR-engineered T cells therapy**. T cells are isolated from patient blood or tumor tissue. TCR α and β chains are then isolated from single T-cell clones and inserted into a lentivirus or retrovirus vector. T cells isolated from the peripheral blood of the patient can be modified with the lentivirus or retrovirus vector to encode the desired TCRαβ sequences. These modified T cells are then expanded *in vitro* to obtain sufficient numbers for treatment and re-infusion back into the patient

Early studies found that TILs isolated from melanoma patients recognized two non-mutated melanoma melanocyte differentiation proteins: MART-1 and gp100 (Kawakami et al., 1994a; Kawakami et al., 1994b). MART-1 and gp100 proteins are often expressed by melanocytes in the skin, eye, and ear. However, many patients who present complete cancer regression did not have a toxic response after treatment with TILs targeting MART-1 or gp100. This demonstrated that it is the antigen-specific T cells in TILs that are crucial for cancer regression. There are however several hurdles to purifying the amount of antigen-specific T cells necessary to be used as a therapy: (1) it is difficult to isolate tumor-specific T cells from many cancer patients; (2) it takes considerable time to obtain a therapeutic amount of tumor-specific T cells. With the introduction of T-cell receptor (TCR) engineering technologies, it became possible to produce antigen-specific T cells. Treatment with engineering, tumor antigen-specific T cells has demonstrated significant clinical successes in patients with metastatic melanoma, colorectal carcinoma, synovial sarcoma, and multiple myeloma (Morgan et al., 2006; Johnson et al., 2009; Parkhurst et al., 2011; Robbins et al., 2011; Rapoport et al., 2015; Robbins et al., 2015) (Fig. 1). Tumor antigen-specific TCR gene-engineered T cells are therefore considered as a potentially “off-the-shelf” treatment for cancer patients.

## THE FOUNDATION OF TCR ACTIVATION

### TCR recognition of pMHC molecules

TCR is expressed on the surface of T cells and consists of two distinct protein chains. In the majority of mature T cells, the TCR consists of α and β chains, although there is a smaller population of T cells in which the TCR consists of γ and δ chains. Antigen recognition by the αβTCR is central to the function of the adaptive immune system. αβTCR bind to the peptide major histocompatibility complex (pMHC) on the surface of antigen-presenting cells. The interaction between an αβTCR and a pMHC is highly specific owing to the fact that T cells are able to distinguish between rare foreign pMHCs and the abundant self pMHC molecules (Germain and Stefanova, 1999). CD8^+^ T cells play an important role in the adaptive immune response in cancer patients and are activated by TCR recognition of specific peptide epitopes. These peptide epitopes are largely generated from endogenous proteins that are presented by MHC class I proteins on the surface of tumor cells (Phan and Rosenberg, 2013). MHC class I proteins are membrane proteins that are expressed on almost all nucleated cells. They are encoded by several families of human leukocyte antigen (HLA-A, B, and C) genes (Brown et al., 2014). Expression of HLA genes can be upregulated by interferon (IFN) signaling, but the expression is often notably down-regulated in tumors. The degree of down-regulation correlates with immune evasion and disease progression in patients with cancer (Agrawal and Kishore, 2000; Leone et al., 2013). T cell activation requires translation of pMHC antigen binding to the TCR and then on to intracellular signaling pathways (Zhang and Bevan, 2011; Obst, 2015; Pageon et al., 2016). *Ex vivo* and *in vivo* studies demonstrate that the dose of antigen presented determines the nature of cytokine expression in T cells (Corse et al., 2011; Tkach et al., 2014).

### TCR signaling transduction

**Figure 2:**
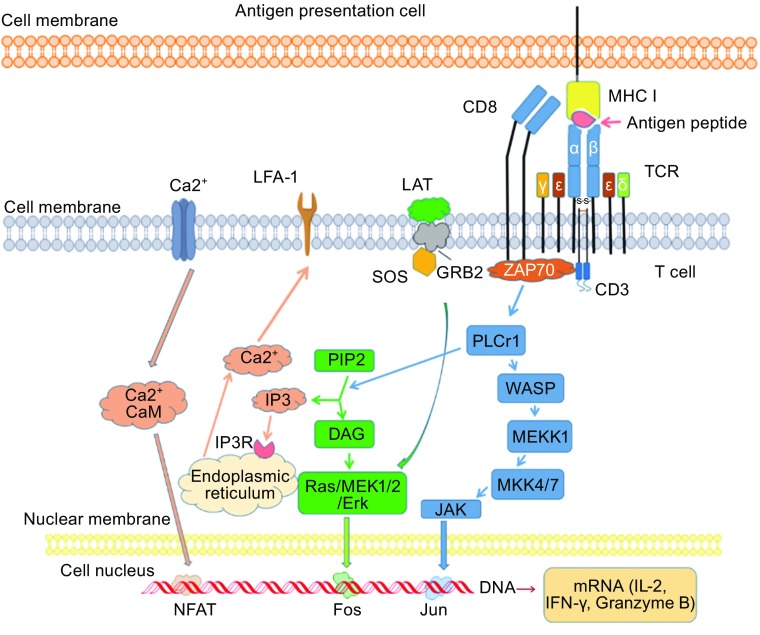
**Schematic demonstration of TCR signaling**. T cells recognize pMHC complexes through the TCR-CD3 cluster. Several classes of proteins are then recruited to the plasma membrane by the activated receptors and participate in signal propagation. Phospholipase C-γ1 (PLC-γ1) cleaves molecules of the membrane phospholipid phosphatidylinositol bisphosphate (PIP2) into inositol triphosphate (IP3) and diacylglycerol (DAG). The interaction of IP3 with its receptors in the endoplasmic reticulum up-regulates the level of Ca^2+^ in the cytosol, further activating the Ca^2+^-binding protein calmodulin. NFAT, as a nuclear factor of activated T cells, is regulated by the calcium pathway. DAG is primarily involved in the activation of the Ras/Erk pathway. T cells are activated though these signaling pathways, releasing IFN-γ, Granzyme B, IL-2, and so on

Naïve T cells undergo clonal expansion of between 10 and 20 rounds of cell division after activation by TCR/pMHC interaction (Zhang and Bevan, 2011; Obst, 2015). Compared with naïve T cells, antigen-stimulated T cells substantially increase antigen responsiveness via a process termed “functional avidity maturation” (Margulies, 2001; Slifka and Whitton, 2001). Studies have found that antigen-stimulated T cells exhibit greater proliferation and cytokine production than naïve T cells (Akbar et al., 1988; Byrne et al., 1988; Sanders et al., 1989; Sallusto et al., 1999). T cells recognize antigen-MHC complexes through the TCR-CD3 cluster. After interaction between the MHC and TCR, several classes of protein are then recruited to the plasma membrane by activated receptors to participate in signal propagation (Fig. 2). Phospholipase C-γ1 (PLC-γ1) cleaves molecules of membrane phospholipid phosphatidylinositol bisphosphate (PIP2), into inositol triphosphate (IP3), and diacylglycerol (DAG). The interaction of IP3 with its receptors in the endoplasmic reticulum upregulates the level of Ca^2+^ in the cytosol, activating the Ca^2+^-binding protein calmodulin. This subsequently regulates nuclear factor of activated T cells (NFAT) proteins. Additionally, DAG activates the Ras/extracellular regulating kinase (Erk) pathway, modulating the nuclear factor Fos. Through all of these interacting signaling pathways, T cells are activated, releasing numerous cytokines and chemokines, including IFN-γ, Granzyme B, and IL-2 (Abraham and Weiss, 2004; Smith-Garvin et al., 2009).

The function of T lymphocytes is largely regulated by TCR signaling. Studies have shown that initial TCR signaling via p38 leads to successive induction of Vitamin D receptor (VDR) and PLC-γ1, both of which are required for classical TCR signaling and T cell activation (von Essen et al., 2010). Genetically blocking TCR internalization inhibits T cell expansion, demonstrating that TCR signaling is required for T cell proliferation. TCR internalization was also required for sustained signaling and activation of key metabolic pathways, including the mechanistic target of rapamycin (mTOR) (Willinger et al., 2015). Acting to control T cell activation, T cell anergy is a tolerance mechanism in which lymphocytes are intrinsically functionally-inactivated following antigen encounter. This phenomenon is often observed in the tumor microenvironment. Zheng et al. found that early growth response protein 2 (Egr2) was necessary for *in vivo* anergy induction when using antigen-induced and tumor-induced anergy models. Egr2 is therefore considered an essential transcriptional regulator of T cell anergy (Zheng et al., 2012). The activation status of T cells and the level of immune response are further controlled by various co-stimulatory (CD28, inducible T cell co-stimulator (ICOS), and OX40) and co-inhibitory (cytotoxic T lymphocyte-associated antigen-4 (CTLA-4), programmed death 1 (PD-1)) molecules (Keir et al., 2008; Chen and Flies, 2013). For example, in the absence of the immune checkpoint protein PD-1, the fusion antibody B7H1-Ig can augment T cell proliferation and immune response. This demonstrates that it is possible to intervene and augment or down-regulate immune response based on B7H1-mediated pathways (Deng et al., 2014).

## APPLICATION OF TCR-ENGINEERED T LYMPHOCYTES

A great deal of scientific studies have shown that TCR-engineered T cells can target and kill cancer cells expressing appropriate antigens (Parkhurst et al., 2011; Cohen et al., 2015; Kageyama et al., 2015; Rapoport et al., 2015; Stronen et al., 2016). However, for treatment to be feasible, it is necessary to first enrich TCR-gene modified antigen specific T cells *in vitro*. Adoptive cell therapy with these engineered cells would be a precise therapy as it targets antigens expressed on cancer cells present in the patient. We will next examine the application of this therapy for the treatment of cancer.

### Selection of an appropriate antigen

Cancer cells can express proteins during development that are different from those found in untransformed cells. Certain antigens that are more frequently expressed by similar tumors are appealing candidates for therapies utilizing immune recognition. These antigens can be unique or shared. Shared antigens are divided into tumor differentiation antigens, over-expressed antigens, and shared tumor-specific antigens (http://cancerimmunity.org/peptide/). Many shared tumor-specific antigens, encoded by cancer-germline genes, have been identified. These antigens can induce an immune responses and are promising candidate targets for use in vaccination or T cell therapy, such as melanoma-associated antigen (MAGE)-A3 (Zhang et al., 2003), MAGE-A4 (Zhang et al., 2002), and New York esophageal squamous cell carcinoma (NY-ESO)-1 (van der Bruggen et al., 1994; Valmori et al., 2000; Van Der Bruggen et al., 2002). Many studies have also reported that tumor differentiation antigens and overexpressed antigens can evoke T cell responses, including MART-1, gp100, carcino-embryonicantigen (CEA), and p53 (Kawakami et al., 1995; Kawashima et al., 1998; Barfoed et al., 2000). In addition to shared antigens, unique antigens also have potential to be used as a targeted treatment. Unique antigens are abnormal proteins that are only expressed by tumor cells. Viral associated antigens found in some cancers can be used to produce antigen-specific T cells, human papilloma virus for example (Draper et al., 2015).

In the past 3 years, neoantigen has garnered much attention as a potential precision immunotherapy. Neoantigens are generated from somatic point mutations in tumor tissues that are absent in normal tissue. Whole genome or exome sequencing can be applied to identify optimal neoantigen candidates for personalized cancer treatment. RNA sequencing can be performed to examine expression and predict whether a neoepitope will be presented by the MHC to be recognized by T cells (Robbins et al., 2013; van Rooij et al., 2013; Brown et al., 2014). In 2016, a study found that mutation-reactive T cells could be enriched from donor-derived T cells and used as an effective therapy for the treatment of patients with metastatic cancer (Prickett et al., 2016; Stronen et al., 2016). CD4^+^ and CD8^+^ T lymphocytes have been shown to target epitopes arising from epigenetic, transcriptional, translational, and post-translational alterations of tumor cells (Coulie et al., 2014). More recently, technological breakthroughs have shown that numerous endogenous mutant cancer proteins are unique to tumor cells. These can be processed into peptides and presented on the surface of tumor cells leading to these cells being recognized *in vivo* as “non-self” or foreign by the immune system. Targeting highly specific neoantigens would enable immune cells to distinguish cancer cells from normal cells and avoid the risk of autoimmunity (Bobisse et al., 2016). Neoantigens therefore represent ideal targets for successful immunotherapy.

Recent exciting results have demonstrated that TILs responding to patient neoantigens can be detected at much higher frequencies than other types (Robbins et al., 2013; van Rooij et al., 2013; Linnemann et al., 2015). Several studies have also found that monoclonal antibodies directed against CTLA-4 are particularly effective at treating cancers with a high burden of somatic mutation (Snyder et al., 2014; Van Allen et al., 2015). In lung and bladder cancer patients treated with pembrolizumab, an antibody targeting PD-1, the non-synonymous mutation burden strongly associates with clinical efficacy (Powles et al., 2014; Rizvi et al., 2015). Isolation and reinfusion of neoantigen-specific T cells may be required to mediate tumor regression without inducing on-target but off-tumor toxicities (Klebanoff et al., 2016). The best currently available technology to obtain large amounts of neoantigen specific T cells is to insert TCR sequences targeting identified neoantigens into T cells *in vitro*. For example, transgenic CD4^+^ lymphocytes that recognize a mutant tumor-specific neoantigen ERBB2 protein induced sustained tumor regression in a patient with cholangiocarcinoma (Tran et al., 2014). These findings indicated that it may be feasible to develop treatments based on the adoptive transfer of TCR-engineered T cells sorted with tetramers bearing mutated epitopes that recognize autologous peripheral T cells (Cohen et al., 2015; Gros et al., 2016). Further interesting results revealed that CD8^+^PD-1^+^ cell populations from PBMCs and TILs had lymphocytes targeting patient specific neoantigens (Gros et al., 2016; Pasetto et al., 2016). However, another study found that the lack of a defined neoantigen resulted in tumor cell resistance in a transplantable tumor model (Matsushita et al., 2012). Whether the neoantigen repertoire in human tumors is stable and therefore consistently targetable is currently unclear. Verdegaal et al. designed a study to observe the landscape of neoantigen dynamics and reveal any detectable stability. Their data demonstrated that specific T cell-recognized neoantigens could be lost by either reduced transcript expression or complete loss of the mutant allele (Verdegaal et al., 2016). Cancer immunotherapy with neoantigen specific T cells should therefore aim to exploit the adaptive capacity of the immune system. Based on these promising results, it may be possible that, in the near future, neoantigen specific T cells could be used as a novel strategy to develop personalized therapies to treat cancer.

Candidate target antigens that are used for TCR-engineered T cell treatment require three features if they are to be utilized: (1) they must be selectively expressed in tumors and not in normal tissues (tumor specificity); (2) they are related to oncogenesis (tumor addiction); (3) they are able to evoke a T-cell response (immunogenicity) (Debets et al., 2016). Broadly speaking, choosing an appropriate antigen is the first and most important step to determine the effectiveness of TCR-engineered T cells.

### Identification of TCR sequences

**Figure 3:**
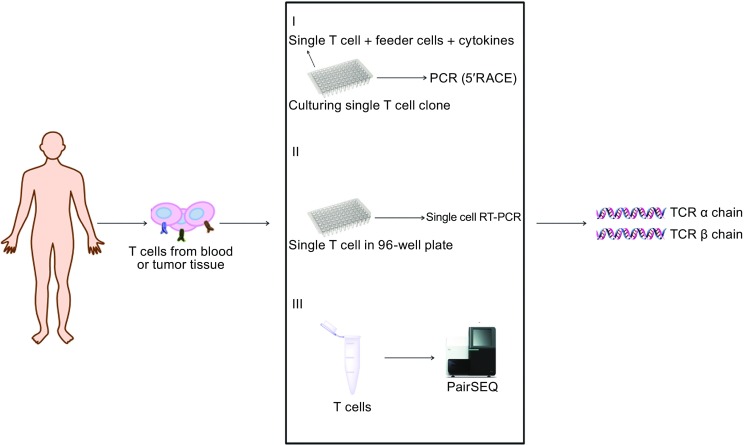
**Three typical procedures to obtain TCR sequence**. Antigen responsive T cells are isolated, specific TCR genes are identified by single clone derived-cDNA sequencing (I), single cell sequencing (II) or paired sequencing of bulk DNA (III)

Identifying TCR sequences is inherently difficult because each T cell contains its own unique TCR that recognizes a distinct set of pMHC molecules. Several methods have been developed to identify TCR sequences from single T cells (Fig. 3). Culturing of T cell clones is the canonical method to identify TCR sequence. Briefly, CD8^+^ or CD4^+^ T cells are purified from peripheral blood mononuclear cells (PBMCs). Serial dilutions allow single T cells being seeded into individual wells of 96-well plates. Finally, these single cells are propagated, generating T-cell clones. The TCR α chain and β chain from these T cell clones are identified and sequenced (Nishimura et al., 1994; Zhang et al., 2010). Using this method, several antigen-specific T cells have been identified that effectively recognize relevant antigens, including tumor antigens. The efficacies of these tumor-specific TCRs to treat cancer have been tested in clinical trials using engineered TCRs (Morgan et al., 2006; Robbins et al., 2015). NY-ESO-1 specific TCR-T cells are the most thoroughly examined and their therapeutic potential has been tested in synovial cell sarcoma, melanoma, and myeloma (Robbins et al., 2011; Rapoport et al., 2015; Robbins et al., 2015).

Recently, single cell RT-PCR and pairSEQ methods have been developed to more rapidly identify TCR sequences. Single cell RT-PCR allows the transcriptomes of thousands of cells to be processed simultaneously (Wu et al., 2014; Redmond et al., 2016). This method can identify the unique TCR and the paired α and β heterodimer of each T cell and has been successfully used to identify paired α and β chains from 91 naïve CD4^+^ T helper cells in mice (Mahata et al., 2014). PairSEQ technology can leverage the diversity of TCR sequences to accurately identify many TCR α and β chain sequences in a single high throughput experiment. Howie et al. used this technology to pair hundreds of thousands of TCR α and β chain sequences from PBMCs isolated from two healthy donors, as well as thousands of sequences from TILs in nine pairs of matched tumor and blood samples (Howie et al., 2015). Pasetto et al. applied PairSEQ technology to identify TCR sequences of T cells derived from 12 fresh metastatic melanomas TIL. This study successfully identified several sequences that showed reactivity against tumor antigens (Pasetto et al., 2016). In summary, PairSEQ is an exciting high throughout technology that can be used to identify the TCR sequences of TILs. This information can then be used to engineer T cells that express antigen specific TCRs. These new technologies have great potential to boost development of TCR-engineered T cell therapy.

### Preclinical studies

Following the identification of tumor antigen-targeting TCRs and their introduction into T cells, it is necessary to perform functional assessment to analyze the sensitivity of T cell responses towards the cognate peptides or autologous tumor cells (Dembic et al., 1986; Kessels et al., 2001). Targeting shared tissue differentiation antigens, such as MART-1, gp100 and CEA, will likely come with the price of toxicity to normal cells in critical organs. It is therefore also necessary to evaluate off-tumor toxicities of TCR-engineered T cells. Although some antigens are not widely expressed, such as the cancer-testis NY-ESO-1 and MAGE families that are expressed on tumor tissue, fetal tissue, and adult testes but not on other normal adult tissues, the safety and affinity of these TCR-engineered T cells should still be assessed. Parkhurst et al. developed a mouse model to isolate CEA-reactive TCRs from splenocytes and perform functional assessment. In this study, they proved that the modified CEA-reactive TCRs were good candidates for future gene therapy and also showed the power of selected amino acid substitutions in the antigen-binding regions of TCR to enhance TCR reactive affinity (Parkhurst et al., 2009). Kunert et al. isolated 10 TCR sequences against four MAGE-C2 epitopes from melanoma patients and designed a set of experiments to evaluate TCR-transgenic T cell function (Kunert et al., 2016). Two MAGE-A3 specific TCRs were isolated from PBMCs of two melanoma patients after MAGE-A3 vaccination. These TCRs recognized MAGE-A3 peptides presented by HLA-DPB1*04:01. The specificity and affinity of these two TCRs were compared and it was found that 6F9 TCR specifically recognized MAGE-A3, but not other members of the MAGE-A family in the context of HLA-DPB1*04:01. The 6F9 TCR was selected for potential TCR gene therapy targeting MHC class II-restricted MAGE-A3 (Yao et al., 2016). An additional issue is that many modified T cells circulating in patients do not have any therapeutic effect because they possess decreased retroviral transgene expression (Kohn et al., 1998). Some studies have reported possible methods to improve TCR gene transfer and to provide a stable system for immunotherapy. Fujio et al. used two independent monocistronic retrovirus vectors to generate ovalbumin (OVA)-specific TCR-T cells. These cells showed a remarkable response to antigen (Fujio et al., 2000). Additionally, a lentiviral vector carrying a bidirectional promoter was used in the Bobisse et al. study. This gene delivery system demonstrated increased transfer efficiency, suggesting lentiviral vectors may be a valid tool for TCR expression in immunotherapy (Bobisse et al., 2009). These preclinical experiments can help guide the application of TCR-T cells in clinical trials but it is essential that new TCRs targeting tumor antigens are tested for their affinity, toxicity, and safety.

### Clinical trials

Adoptive immunotherapy using TCR-engineered T cells has become an important strategy for cancer therapy (Rosenberg and Restifo, 2015) and recent clinical trials have provided encouraging results (Table 1). It was first reported that MART-1 TCR modified lymphocytes could mediate tumor regression in humans in 2006 (Morgan et al., 2006). Clinical trials of MART-1 TCR-engineered T cells in 2009 and 2014 also demonstrated this phenomenon (Johnson et al., 2009; Chodon et al., 2014). Johnson et al. showed that 19% patients treated with gp100 TCR-engineered T cells experience an objective antitumor response (Johnson et al., 2009). In addition to differentiation antigens, clinical trials have also examined cancer-testis antigens, such as MAGE-A3 and NY-ESO-1. In clinical trials using a TCR targeting HLA-A*0201-restricted NY-ESO-1 antigen, objective responses were observed in more than 50% of patients with synovial cell sarcoma, melanoma, and myeloma (Robbins et al., 2011; Rapoport et al., 2015; Robbins et al., 2015). Kageyama et al. conducted a clinical trial examining TCR-modified T cells with a HLA-A*2402-restricted MAGE-A4 in the treatment of esophageal cancer. These TCR-modified T cells could be detected *in vivo* for a prolonged period of time and three patients present minimal tumor lesions for more than 27 months (Kageyama et al., 2015). These clinical trials demonstrate that there can be dramatic tumor regression using TCR-engineered T cells therapy. This has elicited considerable enthusiasm, although it must be noted that most of these clinical trials used only a small number of cancer patients. Additionally, although there has been great progress in adoptive cell therapy with TCR-engineered T cells, some unexpected toxicities have occurred. In a clinic trial using TCR-engineered T cells targeting metastatic colorectal cancer and a high avidity CEA-reactive TCR, all three patients developed severe transient inflammatory colitis due to the TCR reacting to CEA-expressing normal colon epithelium cells (Although one patient had an objective regression of cancer metastatic to the lung and liver) (Parkhurst et al., 2011). In another study, two patients died of cardiogenic shock after infusion with T cells engineered with a TCR against HLA-A*01-restricted MAGE-A3. The artificially modified MAGE-A3 TCR had 4 substitutions in the alpha chain of the CDR2 region and retained the wild type sequences in the beta chain to increase the TCR affinity. This affinity-enhanced TCR may have recognized an epitope derived from an unrelated protein expressed by normal cardiac tissue, but the parental MAGE-A3-specific TCR may also have expanded in the patient without cardiac toxicity through natural thymic selection processes (Linette et al., 2013). A further study also resulted in two patients lapsing into comas and subsequently dying after treatment with autologous MAGE-A3 TCR engineered-T cells. In this study, the modified T cells also recognized an MAGE-A12-derived epitope that was detected in human brain (Morgan et al., 2013). Potential cross-reactivity makes it essential to carefully evaluate the affinity of TCRs and select the appropriate antigens for safe clinical application of TCR-engineered T cells.

**Table 1:** Clinical trials of TCR-engirneering T cells

Antigen	Amino acid sequence of peptide	MHC molecule	Cancer	Number of patients	Year	References
MART-1	AAGIGILTV	HLA-A*0201	Melanoma	17	2006	Morgan et al. (2006)
MART-1	AAGIGILTV	HLA-A*0201	Melanoma	20	2009	Johnson et al. (2009)
gp100	KTWGQYWQV	HLA-A*0201	Melanoma	16	2009	Johnson et al. (2009)
NY-ESO-1	SLLMWITQC	HLA-A*0201	Melanoma	11	2011	Robbins et al. (2011)
			Synovial sarcoma	6		
CEA	IMIGVLVGV	HLA-A*0201	Metastatic colorectal cancer	3	2011	Parkhurst et al. (2011)
MAGE-A3	KVAELVHFL	HLA-A*0201	Metastatic melanoma	7	2013	Morgan et al. (2013)
			Synovial sarcoma	1		
			Esophageal cancer	1		
MAGE-A3	EVDPIGHLY	HLA-A*01	Ulcerated melanoma	1	2013	Linette et al. (2013)
			Myeloma	1		
MART-1	EAAGIGILTV	HLA-A*0201	Metastatic melanoma	14	2014	Chodon et al. (2014)
MAGE-A4	NYKRCFPVI	HLA-A*2402	Esophageal cancer	10	2015	Kageyama et al. (2015)
NY-ESO-1	SLLMWITQC	HLA-A*0201	Multiple myeloma	20	2015	Rapoport et al. (2015)
NY-ESO-1	SLLMWITQC	HLA-A*0201	Synovial cell sarcoma	18	2015	Robbins et al. (2015)
			Melanoma	20		

## IMPROVING THE FUNCTION OF TCR-ENGINEERED T CELLS IN TUMOR MICROENVIRONMENT

Improving the function of TCR-engineered T cells is critical to overcome inhibitory factors within the tumor microenvironment and elicit tumor regression. Many efforts to enhance antigen reactivity and circumvent T cell tolerance have focused on increasing TCR signal strength and generating highly functional T cells. Immune checkpoint proteins, such as PD-1 and CTLA-4, can prevent the activation of T cells in immune system. Blocking the PD-1 pathway has been shown to improve the function of TILs and enhance antitumor immunity (Herbst et al., 2014; Tumeh et al., 2014). It is therefore likely that the PD-1/PD-L1 signaling pathway is a major negative feedback regulator of antigen responsiveness (Okazaki et al., 2013; Honda et al., 2014). Other evidence has implicated PD-1 signaling in modulating the phosphoinositide3-kinase (PI3K), AKT and RAS pathways and cell cycle control (Parry et al., 2005; Patsoukis et al., 2012a; Patsoukis et al., 2012b). TCR-engineered T cells expressing a high level of the inhibitory receptor PD-1 reduced their functional activity (Perez et al., 2015). The efficacy of NY-ESO-1 TCR-engineered T cells was augmented when used in combination with anti-PD-1 antibody (Moon et al., 2016). In addition, cancer cells can influence the local microenvironment by releasing extracellular signals, promoting tumor angiogenesis, and inducing peripheral immune tolerance. Conversely, immune cells in the microenvironment can affect the growth and evolution of cancer cells. Several recombinant cytokines are routinely used in the treatment of cancer, especially IL-2. This cytokine stimulates the growth, differentiation, and survival of antigen-specific T cells and has been used as monotherapy for several different cancer types, including melanoma (Dillman et al., 2012; Vacchelli et al., 2013).

In contrast to the immune checkpoint proteins and immunosuppressive cytokines described above, the number of antigen specific T cells can be affected by chemokines in the tumor microenvironment. Blocking CXCR3, the receptor for the chemokine ligand CXCL9/10, impairs the accumulation of STAT3 deficient CD8^+^ T cells in tumor sites (Yue et al., 2015). Another study demonstrated that the chemokine CXCL10 maintains the effector T cell population (Harris et al., 2012). The chemokine CCL17 produced by CD8α^+^ dendritic cells attracted naïve cytotoxic T cells expressing the chemokine receptor CCR4 (Semmling et al., 2010). Research using chimeric antigen receptor (CAR) transgenic T cells suggests that chemokines enhance the trafficking of T cells to the tumors. CAR-T cells have shown great successes in clinical trials treating leukemia and lymphoma, although there are still issues when using them with solid tumors, such as the low numbers of T cells present at the tumor site. However, the number of GD2-CAR transgenic T cells increased in tumors after co-modification with CCR2b (Craddock et al., 2010). Migration of CAR-T lymphocytes also improved by forced expression of CCR4. The functionality of these cells was not impeded by transgenic expression of CCR4 (Di Stasi et al., 2009). Considering that chemokine receptor-armed CAR-T cells exhibit enhanced tumor infiltration, improving other transgenic TCR-T cell lines with the addition of chemokine receptors may bring better clinical outcomes. In addition, TCR-T cells only recognize intracellular tumor antigens present on the cell surface by MHC molecules, while CAR-T cells can recognize tumor antigens expressed on the tumor-cell surface independent of MHC restriction and antigen processing. A recent study demonstrating the benefits of this approach created CD8^+^ T cells expressing two additional receptors; a gp100 antigen-specific TCR and a melanoma-associated chondroitin sulfate proteoglycan specific CAR (Uslu et al., 2016). These T cells using combined recognition pathways showed greater efficacy by by-passing the mechanisms by which tumor cells escape immune recognition. In conclusion, TCR-engineered T cells therapy, in combination with drugs targeting chemokines, cytokines, and immune checkpoint proteins, may obtain better clinical responses in future treatments.

## FUTURE PROSPECTIVE

There has been considerable progress in adoptive cell therapies using TCR-engineered T cells, a highly personalized cancer therapy. There are still some questions that remain to be answered: (1) How can the inhibitory factors present in the tumor microenvironment be overcome; (2) Is it possible to improve TCR-engineered T cell longevity at the tumor site *in vivo*; (3) Can an effective cocktail of TCR-engineered T cells, including different types of antigen-specific T cells targeting different antigenic epitopes, be identified.

Although some antibodies or recombinant cytokines can be used with TCR-engineered T cells, other currently unidentified factors still exist in tumor microenvironment that may also affect outcome. Another issue is that, once being activated, naïve T cells rapidly proliferate and differentiate into effector T cells and memory T cells after TCR-pMHC interaction. Although these differentiated effector T cells can produce a variety of effector molecules, these cells show high expression of exhaustion markers and rapid progression to cell death. To solve the problem of T cell exhaustion and prolong an effective immune response, some options may be feasible. One such approach is to alter metabolic pathways to enhance engineered T cells persistence. It has been shown that mTOR signals, AMPK-α1 signals, and IL-7 signals support the development of memory CD8^+^ T cells (Rolf et al., 2013; Cui et al., 2015). Based on these observations, it may be necessary to produce long-lived memory-like TCR-engineered T cells expressing metabolic associated molecules.

Verdegaal et al. observed dynamic changes in neoantigens from two patients with stage IV melanoma and found that expression of T cell recognized neoantigens reduced or even lost at the tumor site. This suggests that patients have an improved clinical response if infused with multiple T cell lines with engineered TCRs that recognize different neoantigens (Verdegaal et al., 2016). For example, a recent study demonstrated that T cells engineered with TCRs of the ten most abundant CD8^+^PD-1^+^ clonotypes from a TIL had reactivity against cancer germline antigens and neoantigens (Pasetto et al., 2016). In the future, different types of TCRs may be obtained by culturing CD8^+^PD-1^+^ T cells isolated from patient and could be a novel strategy to develop personalized cancer therapies. T cells co-expressing TCR and CAR also open a new avenue for the design of multifunctional tumor-specific T cells to be used in adoptive transfer (Uslu et al., 2016). For the potential of these therapies to be met, new and accurate approaches will need to be developed.
